# Health Impact Assessment - When Clarity Hinders Implementation

**DOI:** 10.3389/phrs.2024.1607805

**Published:** 2024-11-12

**Authors:** Gabriel Gulis

**Affiliations:** Unit for Health Promotion Research, University of Southern Denmark, Esbjerg Campus, Odense, Denmark

**Keywords:** health promotion, public health, governance, implementation, health impact assessment

I first met with the term “health impact assessment- HIA” at Verona Initiative meeting of WHO EURO in 1999 [1] and found it an extremely relevant method not only for health promotion, but to whole public health. Soon, I learned about Gothenburg Consensus paper [2] and started my journey from Slovakia to Denmark having HIA as a fixed point in my career. European Union funded projects, WHO EURO external consultancy work oriented toward capacity building in a range of European countries, capacity building work in South Korea, international conferences, active participation in development of a HIA section within European Public Health Association all provided me excellent opportunities to learn about HIA, global health, approaches, differences and similarities and I am very grateful for all of that. Yet, I still feel a kind of dissatisfaction, a feeling that we could and should be much further away with implementation and routine use of HIA in practical terms.

There are two key factors leading to this dissatisfaction, both excellently illustrated in the recent paper of Lamprecht et al. [3] and the related commentary by Kim et al. [4]. The first is a persistent lack of clarity on interpretation and understanding of the term HIA documented by both author groups. Despite of efforts to clarify what is HIA and how to interpret it [5–7] there is no common consensus established.

The second factor is related to generally existing research practice gap. Whereas Lamprecht et al. looked at scientific literature, Kim et al. argue that most of HIA papers/reports are done by private sector and as such not identifiable in scientific literature databases. I believe both author groups are right and clearly support the call by Kim et al. for revitalization of databases of HIA case studies. Global institutions such as the WHO or IAIA should consider taking a lead on setting up a database; within European Region of WHO already existing “Environment and Health Hub” at University of Liverpool [8] can be a good starting point.

Based on my experience, allow me to add two more factors; a third factor related to the feeling of slow implementation is hidden in expectations. What do we expect from HIA? How is HIA changing life of population, does it at all? I got this question from municipal health administrators in the city where I live and run a HIA workshop for municipal employees about 20 years ago and I have still hard time to answer it, despite of having been part of the Effectiveness of HIA study several years ago [9]. However, in terms of clarity, this factor opens the question of who is the proper implementing group of HIA? Although HIA by substance is one of tools for public health, a traditional health sector is unlikely to be the one who can implement it. Is it the private sector? As Kim at all argue, yes, likely it is. Or is it a governance sector? If yes, at what level? International, regional, local? Finding answers to these questions is likely to enhance both clarity and implementation.

I cannot leave out a fourth factor contributing to my feeling of dissatisfaction though, I am aware it is a bit provocative one, especially looking inwards into public health research arena. Despite of consistent calls for cross-disciplinary teamwork over time, I often met statements like “I do not work with private consultancy” arguing by potential conflict of interest issues. No question, conflict of interest and other ethical issues must always be considered very seriously but should not be those hindering the use of HIA.

So, paraphrasing the title of the seminal paper by Scott-Samuel [10] “what next HIA”?

First, I would call researchers to provide more quality papers such as the Lamprecht et al. and commentaries as the one by Kim et al! Within the same, and please take this as expression of gratitude to editors of Public Health Reviews who gave us space for this discussion, journal editors, please open for critical discussions even if first reviews are not always positive. Only open and transparent discussion can move any discipline, not only HIA, forward!

Second, within impact assessment society there is a need for open discussion potentially leading to revision of the Gothenburg Consensus Paper. An outcome of such discussion could be an internationally recognized set of quality criteria for HIA, which would make it clear what a HIA is and what an assessment of health impacts is. I believe, the WHO, IAIA and representatives of academia as well as private industry and economic organizations can jointly produce such guidance including all above mentioned factors.
